# Editorial: Biologic drugs in immune-mediated inflammatory diseases, validation, drug-utilization, effectiveness, regulation, costs, and safety in the real-world

**DOI:** 10.3389/fphar.2024.1542453

**Published:** 2025-01-10

**Authors:** Irma Convertino, Luciane Cruz Lopes, Nicole Pratt, Ylenia Ingrasciotta, Marco Tuccori

**Affiliations:** ^1^ Unit of Pharmacology and Pharmacovigilance, Department of Clinical and Experimental Medicine, University of Pisa, Pisa, Italy; ^2^ Sorocaba University, Sorocaba, São Paulo, Brazil; ^3^ Quality Use of Medicines and Pharmacy Research Centre, Clinical and Health Sciences, University of South Australia, Adelaide, SA, Australia; ^4^ Department of Diagnostic and Public Health, Section of Pharmacology, University of Verona, Verona, Italy

**Keywords:** biologic drugs, real-world data, immune-mediated inflammatory diseases, pharmacoepidemiology, safety, cost-effectiveness, DMARDs

Biologic drugs have significantly improved the therapeutic landscape for immune-mediated inflammatory diseases (IMIDs), including rheumatoid arthritis (RA), psoriasis, inflammatory bowel disease (IBD), and systemic lupus erythematosus (SLE) (El-Gabalawy et al., 2010). These targeted therapies, such as monoclonal antibodies and recombinant proteins, have shown remarkable efficacy in clinical trials (Blandizzi et al., 2017), offering hope to patients with chronic inflammatory conditions. However, translating the controlled success of biologics into real-world practice poses challenges (Ferraro et al., 2023), including heterogeneity in patient populations, adherence issues, switching and multiple switches, healthcare resource utilization, and cost concerns (Van Den Bemt et al., 2012; Trifirò et al., 2019; Spini et al., 2024; Convertino et al., 2021; Convertino et al., 2023), highlighted by the increasing prevalence of biological drug users affected by IMIDs over the years (Trifirò et al., 2021). For instance, Ingrasciotta et al. (2024) in the study comparing the characteristics of users of biologics in IMIDs between randomized clinical trials and the real-world setting highlighted that variables such as older age, previous cancer diagnoses and the occurrence of concomitant IMIDs led to the main differences in observations between these two types of investigations (Ingrasciotta et al., 2024).

This Research Topic focused on these challenges by including nine studies that offer valuable insights into the use of biologics in the real-world (Zhang et al.) The studies described below provide a comprehensive overview of drug utilization patterns, treatment outcomes, safety profiles, and economic implications in different healthcare systems across Countries.

Pera et al. conducted a disproportionality analysis using the FDA’s Adverse Event Reporting System (FAERS) to assess the risk of parasitic infections associated with monoclonal antibodies targeting type 2 immune responses (such as biologic drugs used in asthma and eosinophilic disorders). The study revealed significant safety concerns by demonstrating a statistically significant association between certain biologics and the occurrence of parasitic infections, particularly in immunocompromised patients, emphasizing the need for rigorous post-marketing surveillance, especially in vulnerable populations.

Li R. et al. presented a case study of a patient with refractory intestinal Behçet’s disease who was successfully treated with Vedolizumab. This report highlighted the potential of biologics for complex, off-label applications in patients with comorbidities.

Convertino et al. examined disease activity and drug utilization patterns of biologic disease-modifying antirheumatic drugs (bDMARDs) in a cohort of RA patients in the Tuscany region of Italy. This population-based study, which analyzed data from medical records for disease activity information, and a healthcare administrative database, for drug-utilization assessment, showed variability in bDMARD discontinuations driven by patient disease activity and influenced by the clinical guidelines and patient baseline characteristics. The study highlighted the importance of tailored treatment approaches in response to the disease activity.

Zeng et al. reviewed the evolution of anti-TNFα therapies in IBD, from the first-generation originator drugs to the newer biosimilars. Their findings underscored the potential of biosimilars to improve access to treatment while maintaining safety and efficacy, particularly in the management of chronic diseases like IBD. The authors recommended continuous monitoring of biosimilars in real-world practice to confirm their long-term safety and effectiveness.

Li Y. et al. investigated the impact of DMARDs and non-steroidal anti-inflammatory drugs (NSAIDs) on the clinical course of mild-to-moderate COVID-19 in patients with ankylosing spondylitis (AS). Their findings highlighted the need for personalized treatment strategies arising from the pandemic that balance the benefits of controlling AS symptoms with the potential risks of immunosuppressive therapies in the context of viral infections.

Fu et al. examined hepatitis-related adverse events in patients treated with immune checkpoint inhibitors, using data from the FAERS and they identified a significant, albeit relatively rare, risk of hepatitis in these users. The study showed the importance of monitoring liver function for the early detection of adverse events and the effective management of hepatotoxicity.

Vesikansa et al. analyzed healthcare resource utilization in psoriasis patients who received biologic therapies versus those treated with conventional drugs in Finland. The study showed that although biologics are more expensive than conventional treatments, they resulted in lower overall healthcare costs by reducing hospitalizations, emergency visits, and the need for frequent medical consultations. This study provided valuable insights into the economic burden of psoriasis treatment and the potential cost-effectiveness of biologic therapies in real-world practice.

Long et al. performed a systematic review and meta-analysis to evaluate the efficacy and safety of iguratimod, a novel DMARD, in the treatment of inflammation and joint degeneration in RA. The findings highlighted its efficacy and safety in treating both pathologic aspects of RA but highlighted the need for post-marketing monitoring and pharmacoepidemiologic studies to confirm its long-term safety and effectiveness.

Shehab et al. provided real-world data on the effectiveness of biologic therapies in achieving treatment targets in patients with IBD in the Middle East. The study assessed the clinical outcomes of patients treated with anti-TNFα therapies and integrin inhibitors, showing that biologics are effective in controlling disease activity and improving the quality of life for many patients. However, the authors also emphasized the challenges in drug access and adherence to biologics and advocated for improved healthcare infrastructure in their Region.

The studies collected in this Research Topic collectively highlight the importance of real-world data in complementing clinical trial findings, by providing critical insights into the long-term safety, effectiveness, and economic impact of biologics, and by addressing gaps in research. These suggest that future research should prioritize improving pharmacovigilance, increasing accessibility, and integrating personalized medicine. Notably, comprehensive post-marketing surveillance is essential to ensure patient safety, especially in large-scale populations. Biosimilars hold promise for reducing treatment costs, but equitable access to biologics remains a challenge globally and tailoring treatment strategies to individual patient characteristics and disease response can improve outcomes and reduce healthcare burdens.

In conclusion, biologic drugs have undeniably advanced the management of IMIDs, but their utilization in real-world practice requires addressing challenges related to safety, cost-effectiveness, and healthcare accessibility. The findings presented in this Research Topic provide a roadmap for optimizing biologic therapy in several clinical settings. By leveraging real-world evidence, clinicians, researchers, and policymakers can bridge the gap between clinical trials and everyday practice, ensuring better outcomes for patients worldwide.
